# Disruption of the Expression of a Non-Coding RNA Significantly Impairs Cellular Differentiation in *Toxoplasma gondii*

**DOI:** 10.3390/ijms14010611

**Published:** 2012-12-28

**Authors:** Veerupaxagouda Patil, Pamela J. Lescault, Dario Lirussi, Ann B. Thompson, Mariana Matrajt

**Affiliations:** Department of Microbiology and Molecular Genetics, University of Vermont, 95 Carrigan Dr. Burlington, VT 05405, USA; E-Mails: vkpatil13@gmail.com (V.P.); pamela.lescault@uvm.edu (P.J.L.); dlirussi@uvm.edu (D.L.); abthomps@uvm.edu (A.B.T.)

**Keywords:** *T. gondii* development, non-coding RNA, bradyzoites

## Abstract

The protozoan parasite *Toxoplasma gondii* is an important human and veterinary pathogen. Asexual replication of *T. gondii* in humans and intermediate hosts is characterized by two forms: rapidly growing “tachyzoites” and latent “bradyzoite” tissue cysts. Tachyzoites are responsible for acute illness and congenital neurological birth defects, while the more slowly dividing bradyzoite form can remain latent within the tissues for many years, representing a threat to immunocompromised patients. We have developed a genetic screen to identify regulatory genes that control parasite differentiation and have isolated mutants that fail to convert to bradyzoites. One of these mutants has an insertion disrupting a locus that encodes a developmentally regulated non-coding RNA transcript, named *Tg-ncRNA-1*. Microarray hybridizations suggest that *Tg-ncRNA-1* is involved in the early steps of bradyzoite differentiation. Since *Tg-ncRNA-1* does not contain an open reading frame, we used the algorithm Coding Potential Calculator (CPC) that evaluates the protein-coding potential of a transcript, to classify *Tg-ncRNA-1*. The CPC results strongly indicate that *Tg-ncRNA-1* is a non-coding RNA (ncRNA). Interestingly, a previously generated mutant also contains an insertion in *Tg-ncRNA-1*. We show that both mutants have a decreased ability to form bradyzoites, and complementation of both mutants with wild-type *Tg-ncRNA-1* restores the ability of the parasites to differentiate. It has been shown that an important part of bradyzoite differentiation is transcriptionally controlled, but this is the first time that a non-coding RNA is implicated in this process.

## 1. Introduction

*Toxoplasma gondii* (*T. gondii*) is an obligate intracellular protozoan parasite that belongs to the phylum Apicomplexa. It infects a wide variety of intermediate hosts, including humans. *T. gondii* causes congenital hydrocephalus and mental retardation in children, blindness in adults, and encephalitis in immune suppressed patients. This parasite is ubiquitous and the prevalence rate in humans ranges from 15% to 75%, depending on the geography [1,2].

*T. gondii* asexual reproduction consists of two inter-converting developmental stages: rapidly replicating tachyzoites and a slowly growing bradyzoites that form cysts. Whereas tachyzoites are responsible for disease manifestation and are susceptible to drug therapies, bradyzoites evade host immune responses and are resistant to currently available drugs [3,4]. Tachyzoites disseminate quickly throughout the body and differentiate to bradyzoites that can persist within host tissues as dormant cysts, for months or years. In immunocompromised individuals, bradyzoites de-differentiate to tachyzoites, which can cause a life-threatening disease. Hence, the interconversion process between these two developmental stages is crucial for both persistence and disease.

Tachyzoite-to-bradyzoite differentiation can be studied *in vitro* [5–7]. *In vivo*, the critical host molecule that drives this switch is interferon gamma [8,9]. *In vitro* models of differentiation mimic the “stress” of the host immune responses such as treatment with mitochondrial inhibitors, interferon gamma, high pH (8.1), high temperature, nitric oxide, CO_2_ starvation. A significant part of bradyzoite differentiation has been shown to be transcriptionally controlled [9–11]. Despite these advances, little is known about the genes that play a central role in the regulation of this process.

We and others have previously generated differentiation mutants that fail to convert to bradyzoites [11–15]. However, in a few instances, the disrupted loci have been identified and linked to the mutant phenotype. In the present study, we present the characterization of two independently generated bradyzoite differentiation mutants, B7 and 29C3. These two mutants were isolated in two different genetic screens and two different labs, and importantly, they contain insertions disrupting the same transcript. This transcript is predicted to be a non-coding RNA (ncRNA). Complementing these mutants with the wild-type locus restores the ability of the parasites to differentiate. This surprising result suggests that this ncRNA plays an important role in the cellular differentiation of *T. gondii*.

## 2. Results and Discussion

### 2.1. Mutant B7 Is Severely Impaired to Form Bradyzoites

In order to understand the genetic basis and mechanisms underlying bradyzoite differentiation, we have developed a genetic screen to identify regulatory genes that control parasite differentiation, and have isolated mutants that fail to convert to bradyzoites [11,12]. In this screen, we took advantage of parasites that lack an endogenous copy of the HXGPRT gene (useful as both positive or negative selectable marker) and reintroduced this marker under the control of a bradyzoite specific promoter. In this background, following insertional mutagenesis, parasites in which a positive regulator of the differentiation signaling cascade is inactivated are resistant to negative selection, under bradyzoite growth conditions. This selection strategy has allowed us to select the population of parasites that form bradyzoites and recover a population of mutant parasites impaired in their ability to differentiate. One of these mutants (mutant B7) was selected for further analysis.

**Figure 1:**
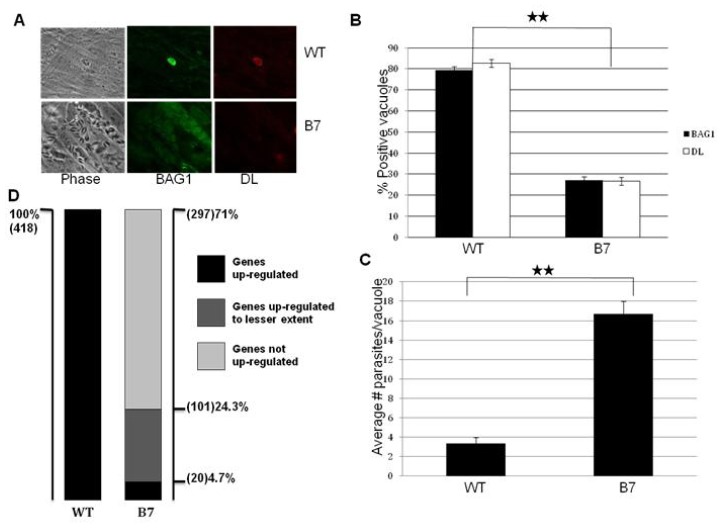
Cellular differentiation is impaired in mutant B7. Confluent HFF cells were infected with the wild-type and mutant parasite lines and subjected to bradyzoite differentiation conditions for 72 h as described in materials and methods. (**A**) Immunofluorescence assay carried out with wild-type (WT) and mutant (B7) parasites after 72 h of bradyzoite growth conditions. Green: bradyzoite antigen 1 (BAG1) [16]. Red: *Dolichos lectin* (DL) [3]; (**B**) The presence/absence of marker expression (BAG1 and DL) was counted for 100 parasite vacuoles, in three independent experiments; (**C**) Proliferation was determined by counting the number of parasites per vacuole (in 100 vacuoles), in three independent experiments; (**D**) Microarray analysis reveals a global defect in the expression of bradyzoite specific genes. The expression of all the genes up-regulated more than two fold in wild-type parasites (total of 418 genes) was analyzed in the mutant B7. Out of these 418 genes, 297 genes are not up-regulated in the mutant parasites; 101 genes are up-regulated but to a lesser extent compared to the expression in wild-type parasites; and 20 genes are up-regulated to the same levels than wild-type parasites. The significance of the data was determined by Student’s *t*-test (* *p* < 0.05, ** *p* < 0.01 and *** *p* < 0.001). The data are represented as mean ± SEM.

Bradyzoites can be distinguished from tachyzoites based on the expression of bradyzoite specific markers and reduced replication rate [4]. We subjected wild-type and mutant parasites to bradyzoite differentiation conditions and determined the expression of bradyzoite specific markers and growth rate (Figure 1). The expression of the major bradyzoite antigen (BAG1) and the cyst wall marker *Dolichos* lectin (DL) is significantly reduced in the mutant B7. After 72 h of *in vitro* bradyzoite induction, ~80% of the wild-type parasites are positive for both BAG1 and DL, while only ~25% of the mutant parasites express these bradyzoite markers (Figure 1A,B).

After invasion of a host cell, *T. gondii* tachyzoites establish an intracellular vacuole where they replicate synchronously with a cell cycle of ~7 h [17]. However, under bradyzoite differentiation conditions, the parasites slow down their replication rate continuously, until they stop replicating in mature cysts. Whereas the tachyzoites completely lyse the host cell monolayer after 72 h of growth, the bradyzoites never lyse the monolayer. Under tachyzoite growth conditions, there is no growth difference between the wild-type and mutant parasites (data not shown). Under bradyzoite differentiation conditions, mutant parasites grow significantly faster, and fail to stop replicating resulting in the lysis of the monolayer. After 72 h, the average number of parasites per vacuole was ~2 and ~16 for the wild-type and B7 mutant respectively (Figure 1C). Taken together, these results show that mutant B7 has a severe defect to form bradyzoites.

In order to gain a better insight of the impact of the insertion on the global gene expression in mutant B7, we carried out microarray hybridizations with the ToxoGeneChip (details of microarray hybridization experiments can be seen in [11]). The data is archived at NCBI GEO under Series GSE23174. Looking at the whole genome, 418 genes are up-regulated more than two fold in the transition from tachyzotes to bradyzoites (Figure 1D). Among these 418 genes up-regulated in wild-type bradyzoites, only 20 genes (4.7%) are up-regulated to the wild-type levels in the mutant B7 (Figure 1D). Another small fraction (24.3%) of bradyzoite specific genes are up-regulated in the mutant, but the induction levels substantially lower than in wild-type parasites. This result suggests that the gene disrupted in the mutant B7, is very likely involved in the early steps of bradyzoite differentiation and, thereby, its loss prevents the expression of a large number of downstream genes.

### 2.2. The Locus Disrupted in Mutant B7 Encodes a Non-Coding RNA

**Figure 2:**
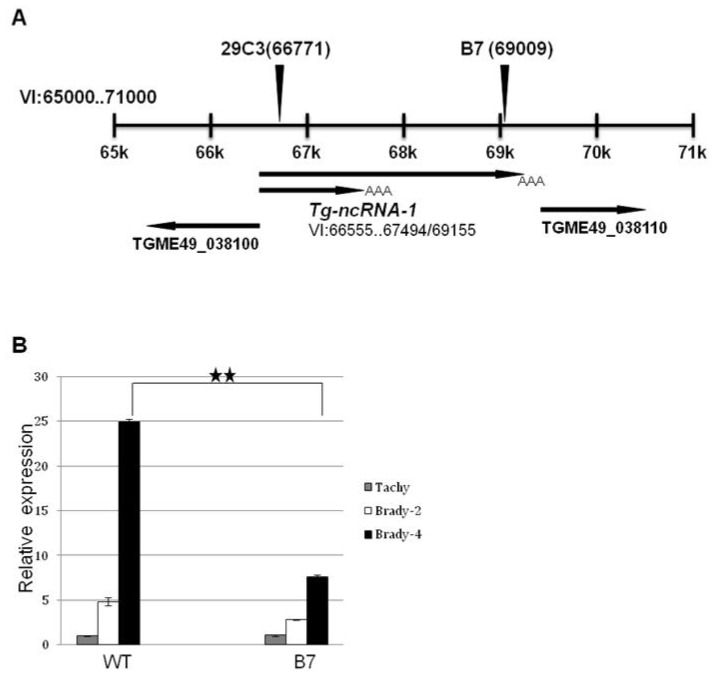
The disrupted locus in mutant B7 encodes a developmentally regulated transcript whose expression in significantly reduced in mutant parasites. (**A**) Schematic representation of *T. gondii* chromosome VI, positions 65,000 to 71,000. The location of the insertion in mutant B7 and mutant 29C3 (described below in the text) is shown. The genes/transcripts present in this region of chromosome VI are also shown; (**B**) The expression of *Tg-ncRNA-1* was examined by Real Time PCR. Total RNA was extracted from wild-type (WT) and mutant (B7) under tachyzoite growth conditions (Tachy), 2 days and 4 days after bradyzoite induction (Brady-2 and Brady-4 respectively). The values indicate the relative gene expression levels normalized to the expression levels of α-tubulin (constitutively expressed gene-endogenous control). The significance of the data was determined by Student’s *t*-test (* *p* < 0.05, ** *p* < 0.01 and *** *p* < 0.001). The data are represented as mean ± SEM. The expression of TGME49_038100 and TGME49_038110 is not affected in the mutant parasites or developmentally regulated.

Southern blot analysis shows that the B7 mutant has been disrupted in a single locus, and we identified the genomic DNA flanking the insertion site using a combination of plasmid rescue and inverse PCR [11]. Mutant B7 has an insertion on chromosome VI, which is 345 bp upstream of TGME49_038110, a gene predicted to be a replication factor (Figure 2A). We examined the transcripts expressed from this locus using rapid amplification of cDNA ends (RACE). Using this approach, we discovered that the insertion is directly disrupting a transcript that has no gene prediction in ToxoDB, which is not surprising since it does not contain an open reading frame. RACE also shows that this gene is alternatively spliced, producing two transcripts, one is 2601 bp long and the other is 940 bp long. This gene was named *Tg-ncRNA-1* (Figure 2A).

**Figure 3:**
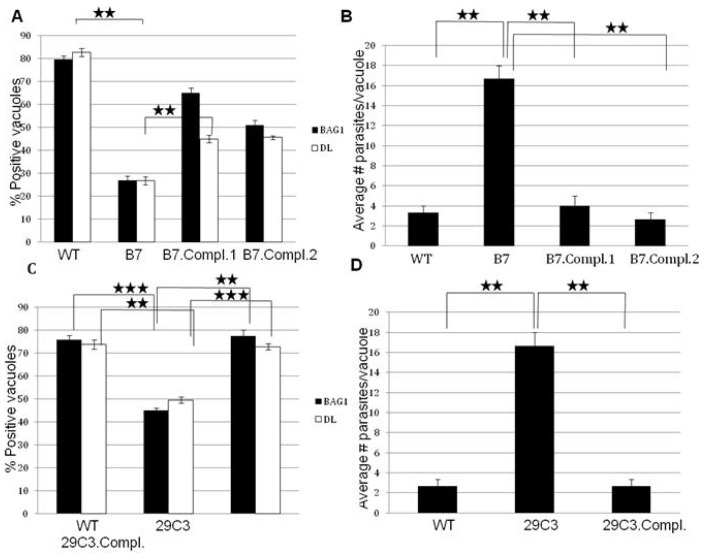
Complementation of mutants B7 and 29C3 rescues the wild-type phenotype. Confluent HFF cells were infected with the wild-type (WT), mutant (B7 or 29C3), and complemented parasite lines (B7.Compl.1 or B7.Compl.2 or 29C3.Compl); and subjected to bradyzoite differentiation conditions for 72 h. (**A**) and (**C**) The presence/absence of bradyzoite marker expression (BAG1 and DL) was counted for 100 parasite vacuoles, in three independent experiments. (**B**) and (**D**) Proliferation was determined by counting the number of parasites per vacuole (in 100 vacuoles), in three independent experiments. The significance of the data was determined by Student’s *t*-test (* *p* < 0.05, ** *p* < 0.01 and *** *p* < 0.001). The data are represented as mean ± SEM.

Real-time PCR shows that *Tg-ncRNA-1* is developmentally regulated; its expression is up-regulated 24 fold in the transition from tachyzoites to bradyzoites in wild-type parasites (Figure 2B). In the mutant bradyzoites, *Tg-ncRNA-1* expression is significantly reduced, ~3 fold reduced (Figure 2B). We examined the expression of the other genes that are close to the insertion site, and none of these genes are either affected in the mutant parasites or developmentally regulated (data not shown). These results strongly suggest that the gene responsible for the mutant phenotype is *Tg-ncRNA-1*, which was confirmed by complementation assays (see below, Figure 3).

Since *Tg-ncRNA-1* does not contain an open reading frame, we suspected that this gene encodes a non-coding RNA (ncRNA). The basis of the classification of a transcript as ncRNA, are extensively discussed in [18]. To evaluate if *Tg-ncRNA-1* is indeed a ncRNA, we used the algorithm CPC (Coding Potential Calculator), which evaluates the protein-coding potential of a transcript based on six biologically meaningful sequence features [19]. The results obtained after running CPC are shown in Table 1. In addition of running the sequence of *Tg-ncRNA-1,* for comparison, we ran two *T. gondii* protein-coding sequences (TGGT1_087710 and TGME49_000320); and two mouse sequences, one classified as ncRNA (Tsix_mus) and one mouse protein-coding gene (AF282387).

The supporting evidence for the classification of each transcript is shown in the last six columns of Table 1. These six columns show the six features used to distinguish coding from non-coding. A detailed description of each feature can be found at the CPC server [19]. Briefly, a true protein-coding transcript is likely to have more hits with known proteins than an ncRNA, and the hits are likely to have higher quality. The hit number and hit score are measures of these features. Also, for a true protein-coding transcript most of the hits are likely to reside within one frame, and the frame score measures this. The higher the hit score and frame score, the better overall quality of the hits and the more concentrated the hits are in one frame, and therefore, it is more likely that the transcript is protein-coding. The last three features in Table 1 assess the extent and quality of the ORF. A large coverage of the predicted ORF is an indicator of good ORF quality. The Log-odds score is also an indicator of the quality of a predicted ORF, and the higher the score, the higher the quality. Lastly, “type” measures the integrity of the predicted ORF, which indicates if an ORF begins with a start codon and ends with an in-frame stop codon. These six features taken together were used to calculate the coding potential score (Table 1). The further away the coding potential score is from zero, the more reliable the prediction is. In general, scores that are smaller than −1 or bigger than 1 are significant, and transcripts with a score between −1 and 1 are classified by CPC as “weak non-coding” or “weak coding” [17]. These results strongly indicate that *Tg-ncRNA-1* is a ncRNA.

**Figure 4:**
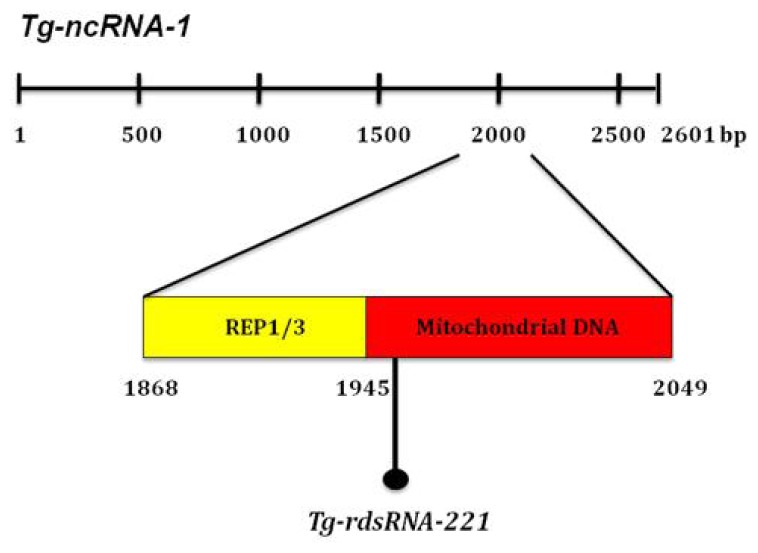
*Tg-ncRNA-1* encodes a small RNA that belongs to the REP-derived (rdsRNA) familiy. Schematic representation of *Tg-ncRNA-1* long isoform, 2601 bp (isoforms shown in Figure 2A). *Tg-ncRNA-1* contains a region with high similarity to the *T. gondii* mitochondrial-like DNA sequences REP1 and REP3 (REP1/3—yellow), and a region with high similarity to *Plasmodium* parasites mitochondrial DNA (mitochondrial DNA—red). The numbers indicate the nucleotide positions within *Tg-ncRNA-1*. The location of the 24-mer rdsRNA is indicated (*Tg-rdsRNA-221*).

The silencing machinery of *T. gondii*, which comprises a big diversity of plant-like and metazoan-like small RNAs (sRNAs), has been characterized [20]. Among these diverse sRNAs, there is a group named *Toxoplasma* REP-derived sRNAS (rdsRNAs) [20]. The rdsRNAs are generated from the repetitive elements REP1 (961 bp), REP2 (1099 bp) and REP3 (1884 bp), which are mitochondrial-like sequences dispersed throughout the nuclear genome of *T. gondii* [21]. Braun and co-workers suggested that the REP elements are the precursors required for rdsRNA synthesis [18]. Sequence analysis showed that *Tg-ncRNA-1* (2601 bp) contains a region (positions 1868–1945 within *Tg-ncRNA-1*) with high similarity to the *T. gondii* mitochondrial-like DNA sequences REP1 and REP3. Immediately downstream of these repeats (positions 1967–1991) *Tg-ncRNA-1* encodes a 24-mer that belongs to a family of small RNAs cloned and sequenced by Braun *et al*., family 221/ncRNA S33 (Figure 4). This 24-mer is also included in a region (positions 1945–2049) with high similarity to *Plasmodium* parasites mitochondrial DNA. Taken together, these findings strongly suggest that *Tg-ncRNA-1* encodes a small RNA that belongs to the rdsRNA familiy, here designated *Tg-rdsRNA-221* (Figure 4). More experiments need to be done to determine if *Tg-rdsRNA-221* behaves functionally as its precursor *Tg-ncRNA-1.* These experiments will be experimentally challenging, due to it is very difficult to obtain large quantities of RNA from bradyzoites. Of note, the *T. gondii* silencing machinery was characterized under tachyzoite conditions only [20], but there are no reports of the bradyzoite silencing machinery. Braun *et al.* showed that the REP elements can encode more than one rdsRNA. Therefore, it is also possible that, in addition to *Tg-rdsRNA-221*, *Tg-ncRNA-1* generates other bradyzoite specific rdsRNAs.

### 2.3. Another Mutant (29C3) That Exhibits a Defect in Bradyzoite Differentiation also Has an Insertion within the Gene Tg-ncRNA-1

We obtained another insertional mutant, named 29C3 (generated by Signature Tagged Mutagenesis), and its corresponding wild-type parental line (named C3), from Laura Knoll’s lab [22]. The 29C3 mutant showed a reduced number of cysts in an *in vivo* mouse model, and it was selected in a screen designed to isolate avirulent *T. gondii* mutants [20]. We were interested in analyzing 29C3 because it contains an insertion within the *Tg-ncRNA-1* transcript. In 29C3, the insertion is located at a different position, compared to mutant B7 (Figure 2A). Therefore, we examined if 29C3 also shows bradyzoite differentiation defects *in vitro*.

**Figure 5:**
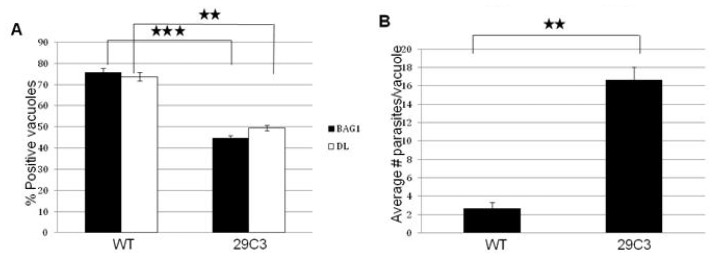
Another independent insertional mutant (29C3) containing an insertion in *Tg-ncRNA-1* is defective in bradyzoite differentiation. Confluent HFF cells were infected with the wild-type (WT) and mutant parasites (29C3) and subjected to bradyzoite differentiation conditions for 72 h. (**A**) The presence/absence of bradyzoite marker expression (BAG1 and DL) was counted for 100 parasite vacuoles, in three independent experiments; (**B**) Proliferation was determined by counting the number of parasites per vacuole (in 100 vacuoles), in three independent experiments. The location of the insertion in 29C3 is shown in Figure 2A. The significance of the data was determined by Student’s *t*-test (* *p* < 0.05, ** *p* < 0.01 and *** *p* < 0.001). The data are represented as mean ± SEM.

We subjected 29C3 mutant to bradyzoite differentiation condition in the same way we did for mutant B7. We determined the expression of bradyzoite specific markers and growth profile. We investigated the expression of the major bradyzoite antigen, BAG1 and the presence of *Dolichos biflorus* lectin staining, a marker that binds to the cyst wall. After 72 h of *in vitro* bradyzoite induction, the mutant 29C3 shows a reduced expression of the bradyzoite markers (Figure 5A). The growth rate is also significantly affected in the mutant (Figure 5B). These results suggest that the mutant 29C3 is defective in bradyzoite differentiation.

The fact that two independent mutants, B7 and 29C3, with an insertion within the gene *Tg-ncRNA-1*, exhibit an important defect in bradyzoite differentiation, strongly suggests that this gene plays a significant role in cyst formation.

### 2.4. Complementation of Mutants B7 and 29C3 Restores the WT Phenotype

We used a cosmid (PSBLS72) that spans the insertion sites of both B7 and 29C3 mutants to complement both of them. A cosmid was used in order to capture all possible genomic regulatory regions flanking *Tg-ncRNA-1*. Stable parasite lines expressing wild-type *Tg-ncRNA-1*, were obtained by transfecting the cosmid PSBLS72 and selecting with phleomycin [23,24].

These complemented parasite lines were subjected to bradyzoite differentiation conditions to characterize them functionally (Figure 3). The expression of bradyzoite markers increases significantly in the complemented B7 and 29C3 mutants (Figure 3A,C), and the growth rate returns to wild-type levels (Figure 3B,D). The cosmid used for complementation contains a few other genes in addition to *Tg-ncRNA-1,* but this gene is the only one within the cosmid that has altered expression levels in the mutant parasites; wild-type parasites up-regulate this gene ~24 fold after bradyzoite induction while it’s expression is significantly reduced in the mutant bradyzoites, ~3 fold reduced (Figure 2). The other genes present in this cosmid show unaffected expression. These results indicate that *Tg-ncRNA-1* is responsible for the phenotype of mutants B7 and 29C3, and complementation with the wild-type locus restores the parasite’s ability to form bradyzoites.

## 3. Experimental Section

### 3.1. Parasite Growth and Differentiation

All the parasites were maintained by serial passage in the confluent Human foreskin fibroblast (HFF) cells as described previously [25]. The parasites were subjected to low CO_2_ condition leading to pyrimidine starvation [12]*in vitro* to induce bradyzoite differentiation. The conditions were achieved by using the Minimum essential medium without NaHCO_3_ but supplemented with 25 mM HEPES [12].

### 3.2. Immunofluorescence Assays

HFF cells were grown to confluence on glass coverslips in six well plates (Becton Dickinson, Franklin Lakes, NJ, USA). The confluent HFF cells were infected with equal number of mutant and the parental wild-type (WT) parasites. After 72 h of bradyzoite induction by CO_2_ starvation, the coverslips were fixed with 3.7% formaldehyde in phosphate-buffered saline (PBS) for 10 min at room temperature, permeabilized with 0.25% Triton-X-100 in PBS for 15 min at room temperature and blocked with 1× PBS 1% bovine serum albumin (BSA) for 30 min at room temperature. The coverslips were incubated for 1 h at room temperature, with antibody against BAG1 diluted 1:100 in 1× PBS 1% BSA. The coverslips were washed three times and were incubated with a secondary antibody 488 (goat anti-rabbit immunoglobulin G (IgG) conjugated to Alexa fluor 594) (Molecular Probes, Eugene, OR, USA) diluted 1:1000 and tetramethyl rhodamine isothiocyanate (TRITC)-labelled *Dolichos biflorus* lectin (Sigma-Aldrich, St. Louis, MO, USA) diluted 1:50, for 1 h at room temperature. The coverslips were washed three times, dipped in distilled water, blotted and mounted with Fluoromount-G (SouthernBiotech, Birmingham, AL, USA). The slides were analysed using a Leica DMIRE2 fluorescence microscope (Leica Microsystems Inc., Chatsworth, CA, USA) and images were captured using Improvision Openlab 4.0.2 software (Improvision, Coventry, UK). The samples were examined at 100× magnification and 100 vacuoles were counted. The number of vacuoles positive for BAG1 and DL staining were expressed as % positive vacuoles from duplicate experiments. The number of parasites/vacuole was recorded simultaneously and the average number of parasites/vacuole was determined.

### 3.3. Transfection and Bleomycin (BLE) Selection

Approximately 25–30 μg cosmid DNA with BLE cassette/transfection was resuspended in the required amount of sterile milliQ water to a final volume of 100 μL and 10 μL Sodium Acetate (3M) was added. Absolute ethanol (250 μL) was added to the sample and the sample was frozen at −80 °C for at least 10 min. DNA precipitation was carried out by centrifuging the sample at maximum speed for 15 min at RT. The DNA sample was washed with 1 mL 70% ethanol with two 10 min spins. The precipitated cosmid DNA was resuspended in 100 μL of cytomix (0.015 g ATP and 0.019 g glutathione were added fresh to 12.5 mL of Cytomix for each transfection). Freshly lysed out parasites were harvested and syringe filtered to remove the cell debris. The parasites were counted and spun down. The parasites were resuspended in appropriate volume of the cytomix, to obtain approximately 10^7^ parasites/mL.

Cosmid DNA (100 μL) was mixed with parasites (300 μL) into 2 mm gap cuvettes. Parasites were electroporated at 1500 V, 25 Ω, 25 μF, and were added to fresh T25 flasks with fresh ED1 without Phleomycin and incubated at 37 °C. The parasites were harvested after complete lysis, spun down and resuspended in ED1 with 50 μg/mL phleomycin. Parasites were then subjected to extracellular BLE selection by incubating at 37 °C in a waterbath for four hours in 4 mL medium. The parasites were spun down and resuspended in fresh ED1 with 5 μg/mL phleomycin. This sample of parasites was split into two equal halves and were plated into two T25 flasks for intracellular BLE selection. After complete lysis, the parasites were subjected to two more rounds of extracellular and intracellular BLE selection. The resistant parasites were cloned out by limiting dilution and were subjected to appropriate functional analysis.

### 3.4. Quantitative Real Time RT PCR (TaqMan)

Indicated mutant and parental WT parasite lines were cultured under tachyzoite and bradyzoite differentiation conditions for 72 h. At the end of incubation, total RNA was extracted using RNeasy kit (Qiagen Sciences, Germantown, MD, USA). RNA (2 μg) was subjected to DNase (Invitrogen, Carlsbad, CA, USA) treatment for 45 min at room temperature. At the end of treatment, 1 μL of 25 mM EDTA was added to the reaction mixture and subjected to denaturation at 65 °C for 15 min. Further, 1 μL of the DNase treatment mixture was aliquoted and mixed with 6 μL DEPC water and used as DNA contamination control later. The recipe used for DNase treatment comprised of RNase inhibitor (RNasin, Promega, Madison, WI, USA) 0.5 μL, DNase buffer (Invitrogen, Carlsbad, CA, USA) 1.0 μL, DNase I, 122.5 U/μL (Invitrogen, Carlsbad, CA, USA) 1.0 μL. Volume was made to 10 μL with DEPC water (GENEMate, Irvine, CA, USA).

DNase treated RNA (9 μL) was mixed with 19 μL of coktail (described below) and subjected to RT reaction (Program A6: RD, Thermal Hybaid cycler, Thermo Fisher Scientific Inc. Waltham, MA, USA). The thermal cycler conditions used for the RT reaction were as follows: 25 °C for 10 min, 42 °C for 60 min and 70 °C for 15 min. The cDNA thus generated was stored at −20 °C. Undiluted cDNA samples were used for all TaqMan experiments. The recipe used for the RT reaction consisted of DEPC water (GENEMate, Irvine, CA, USA) 2.8 μL, 5× RT buffer (Invitrogen, Carlsbad, CA, USA) 6.0 μL, dNTPs (10 mM, Promega, Madison, WI, USA) 2.0 μL, Random Primers, 50 ng/μL (Promega, Madison, WI, USA) 3.0 μL, RNase inhibitor 40 U/μL (RNAsin, Promega, Madison, WI, USA) 1.0 μL, DTT 0.1 M (Invitrogen, Carlsbad, CA, USA) 3.0 μL, SuperScript II, 200 U/μL (Invitrogen, Carlsbad, CA, USA) 1.2 μL.

The primer and probe sets (Sigma Genosys, Haverhill, UK) for the candidate genes were designed using Primer Express software, version 3.0 (Applied Biosystems, Foster City, CA, USA). Real-time quantitative PCR (qPCR) was performed using a 7500 Fast Real-Time PCR system (Applied Biosystems, Foster City, CA, USA) with a reaction mixture volume of 20 μL containing TaqMan Fast Universal PCR 2× Master Mix (Applied Biosystems, Foster City, CA, USA), 900 nM of each primer, 200 nM of each probe and 1 μL of cDNA. The reaction conditions were 95 °C for 20 s, followed by 40 cycles each of 95 °C for 3 s and 60 °C for 30 s. No template controls were run with every experiment and all determinations were performed in duplicates. The relative gene expression levels were calculated as the fold change using the formula 2^−ΔΔ^*^CT^*, where ΔΔ*CT* = ΔΔ*C*_T_ of the target genes − Δ*C*_T_ of the calibrator and Δ*C*_T_ = average target *C*_T_ value − average endogenous control *C*_T_ value. The constitutively expressed gene DHFR was used as the endogenous control.

## 4. Conclusions

Tachyzoite-bradyzoite interconversion plays a critical role in the pathogenesis of *T. gondii* and the genetic mechanisms that drive this process are not clearly understood [1,4]. We and others have generated bradyzoite differentiation mutants to understand the genetic basis of cellular differentiation in *T. gondii* [11–15,22]. In this report, we describe the characterization of two of these mutants with insertions disrupting the same transcript: *Tg-ncRNA-1*. The Coding Potential Calculator (CPC) strongly suggests that *Tg-ncRNA-1* encodes a non-coding RNA.

This is the first time that a ncRNA has been linked to bradyzoite differentiation in *T. gondii*. A growing number of studies in different species show that ncRNAs have important regulatory functions, and in many cases are associated with cell differentiation and development [18,26,27]. NcRNAs are classified as long ncRNAs (>200 nucleotides), or short ncRNAs (<200 nt and typically ~20–30 nt long). Many long ncRNAs mediate epigenetic changes recruiting chromatin remodeling complexes [18,28]. Histone modifying complexes have been linked to differentiation in *T. gondii* [10]. Behnke and co-workers demonstrated that conventional promoter mechanisms work in concert with the chromatin-remodeling machinery to regulate bradyzoite gene expression [10]. However, the mechanism by which the chromatin-remodeling machinery is recruited to the specific genomic loci is not clearly understood. Emerging research suggests that ncRNAs provide the specificity to the genomic loci and it is quite possible that ncRNAs function in concert with chromatin-remodeling complexes to reprogram the gene expression during tachyzoite to bradyzoite differentiation. Hence, it is tempting to implicate *Tg-ncRNA-1* in this process, but more work remains to be done to test this hypothesis.

Long ncRNAs are sometimes precursors of small RNAs. The *T. gondii* genome has good RNAi gene candidates and yet the efficient use of RNAi for specific gene silencing is elusive [29]. Braun and coworkers reported the characterization of the tachyzoite RNA silencing machinery and they did an exhaustive analysis of the small *T. gondii* RNAome [20]. This RNAome comprises metazoan-like microRNAs (miRNA), and plant-like repeat associated small RNAs (rdsRNA) and satellite associated RNAs (satRNA) [20]. All these RNAs bind to the *T. gondii* homolog of argonaute (TgAgo). MiRNA-loaded TgAgo associates with polysomes, which likely regulates translation. TgAgo also co-purifies with chromatin-repressing complexes, which suggests a role in transcriptional silencing, most likely through association with rdsRNAs or satRNAs [20]. Our results suggest that *Tg-ncRNA-1* may be the precursor of a small RNA that belongs to the rdsRNA family. Therefore, another exciting hypothesis is that rdsRNA members are involved in transcriptional gene silencing during bradyzoite differentiation.

This report provides the first evidence for a direct link between the impaired bradyzoite differentiation phenotype and a non-coding RNA gene. To date, many long and small non-coding RNAs have been sequenced and characterized in many species, but finding a phenotype associated to a ncRNA has not been straightforward. This work opens up an exciting area of research on uncovering the mechanisms by which *Tg-ncRNA-1* regulates tachyzoite to bradyzoite differentiation.

## Figures and Tables

**Table 1:** The coding potential calculator (CPC) shows that *Tg-ncRNA-1* is a non-coding RNA. B41(L) and B41(S) correspond to *Tg-ncRNA-1* long isoform and short isoform respectively (isoforms shown in Figure 2A).

Sequence ID	C/NC	Coding potential score	Hit num	Hit score	Frame score	Coverage	Log-odds score	Type
B41(L)	Noncoding	−1.4067	0	0.0	0.0	8.07%	20.32	Full
B41(S)	Noncoding	−1.2738	0	0.0	0.0	22.34%	20.32	Full
TGGT1_087710 cGMP kinase	Coding	2.69204	250	49.54	514.4	3.73%	80.42	Partial
TGME49_000320 HXGPRT	Coding	1.39359	140	17.73	128.9	12.41%	62.36	Partial
Tsix_mus	Noncoding	−1.3004	0	0.0	0.0	3%	27.5	Full
AF282387	Coding	3.32462	150	25.00	208.4	99.43%	109.4	Full

## References

[1] Wong S.Y., Remington J.S. (1993). Biology of *Toxoplasma gondii*. AIDS.

[2] Montoya J.G., Liesenfeld O. (2004). Toxoplasmosis. Lancet.

[3] Boothroyd J.C., Black M., Bonnefoy S., Hehl A., Knoll L.J., Manger I.D., Ortega-Barria E., Tomavo S. (1997). Genetic and biochemical analysis of development in *Toxoplasma gondii*. Philos. Trans. R. Soc. Lond. B.

[4] Weiss L.M., Kim K. (2000). The Development and biology of bradyzoites of *Toxoplasma gondii*. Front. Biosci.

[5] Soête M., Camus D., Dubremetz J.F. (1994). Experimental induction of bradyzoite-specific antigen expression and Cyst formation by the RH strain of *Toxoplasma gondii in vitro*. Exp. Parasitol.

[6] Soete M., Fortier B., Camus D., Dubremetz J.F. (1993). *Toxoplasma gondii*: Kinetics of bradyzoite-tachyzoite interconversion *in vitro*. Exp. Parasitol.

[7] Weiss L.M., Laplace D., Takvorian P.M., Tanowitz H.B., Cali A., Wittner M.A. (1995). Cell culture system for study of the development of *Toxoplasma gondii* bradyzoites. J. Eukaryot. Microbiol.

[8] Suzuki Y., Orellana M.A., Schreiber R.D., Remington J.S. (1988). Interferon-gamma: The major mediator of resistance against *Toxoplasma gondii*. Science.

[9] Subauste C.S., Remington J.S. (1991). Role of gamma interferon in *Toxoplasma gondii* infection. Eur. J. Clin. Microbiol. Infect. Dis.

[10] Behnke M.S., Radke J.B., Smith A.T., Sullivan W.J., White M.W. (2008). The Transcription of bradyzoite genes in *Toxoplasma gondii* is controlled by autonomous promoter elements. Mol. Microbiol..

[11] Lescault P.J., Thompson A.B., Patil V., Lirussi D., Burton A., Margarit J., Bond J., Matrajt M. (2010). Genomic data reveal *Toxoplasma gondii* differentiation mutants are also impaired with respect to switching into a novel extracellular tachyzoite state. PLoS One.

[12] Matrajt M., Donald R.G., Singh U., Roos D.S. (2002). Identification and characterization of differentiation mutants in the protozoan parasite *Toxoplasma gondii*. Mol. Microbiol.

[13] Singh U., Brewer J.L., Boothroyd J.C. (2002). Genetic analysis of tachyzoite to bradyzoite differentiation mutants in *Toxoplasma gondii* reveals a hierarchy of gene induction. Mol. Microbiol.

[14] Anderson M.Z., Brewer J., Singh U., Boothroyd J.C. (2009). A pseudouridine synthase homologue is critical to cellular differentiation in *Toxoplasma gondii*. Eukaryot. Cell.

[15] Vanchinathan P., Brewer J.L., Harb O.S., Boothroyd J.C., Singh U. (2005). Disruption of a locus encoding a nucleolar zinc finger protein decreases tachyzoite-to-bradyzoite differentiation in *Toxoplasma gondii*. Infect. Immun.

[16] Bohne W., Gross U., Ferguson D.J., Heesemann J. (1995). Cloning and characterization of a bradyzoite-specifically expressed gene (hsp30/bag1) of *Toxoplasma gondii*, related to genes encoding small heat-shock proteins of plants. Mol. Microbiol.

[17] Fichera M.E., Bhopale M.K., Roos D.S. (1995). *In vitro* assays elucidate peculiar kinetics of clindamycin action against *Toxoplasma gondii*. Antimicrob. Agents Chemother.

[18] Matrajt M. (2010). Non-coding RNA in apicomplexan parasites. Mol. Biochem. Parasitol.

[19] Kong L., Zhang Y., Ye Z.Q., Liu X.Q., Zhao S.Q., Wei L., Gao G. (2007). CPC: Assess the protein-coding potential of transcripts using sequence features and support vector machine. Nucleic Acids Res.

[20] Braun L., Cannella D., Ortet P., Barakat M., Sautel C.F., Kieffer S., Garin J., Bastien O., Voinnet O., Hakimi M.A. (2010). A complex small RNA repertoire is generated by a plant/fungal-like machinery and effected by a metazoan-like argonaute in the single-cell human parasite *Toxoplasma gondii*. PLoS Pathog.

[21] Ossorio P.N., Sibley D.L., Boothroyd J.C. (1991). Mitochondrial-like DNA sequences flanked by direct and inverted repeats in the nuclear genome of *Toxoplasma gondii*. J. Mol. Biol.

[22] Frankel M.B., Mordue D.G., Knoll L.J. (2007). Discovery of parasite virulence genes reveals a unique regulator of chromosome condensation 1 ortholog critical for efficient nuclear trafficking. Proc. Natl. Acad. Sci. USA.

[23] Messina M., Niesman I., Mercier C., Sibley L.D. (1995). Stable DNA Transformation of *Toxoplasma gondii* using phleomycin selection. Gene.

[24] Soldati D., Kim K., Kampmeier J., Dubremetz J.F., Boothroyd J.C. (1995). Complementation of *Toxoplasma gondii* ROP1 knock-out mutant using phleomycin selection. Mol. Biochem. Parasitol.

[25] Roos D.S., Donald R.G., Morrissette N.S., Moulton A.L.C. (1995). Molecular tools for genetic dissection of the protozoan parasite *Toxoplasma gondii*. Methods Cell Biol.

[26] Mattick J.S. (2007). A new paradigm for developmental biology. J. Exp. Biol.

[27] Amaral P.P., Mattick J.S. (2008). Non-coding RNA in development. Mamm. Genome.

[28] Khalil A.M., Guttman M., Huarte M., Garber M., Raj A., Rivea Morales D., Thomas K., Presser A., Bernstein B.E. (2009). Many human large intergenic non-coding RNAs associate with chromatin-modifying complexes and affect gene expression. Proc. Natl. Acad. Sci. USA.

[29] Ullu E., Tschudi C., Chakraborty T. (2004). RNA interference in protozoan parasites. Cell. Microbiol.

